# Increasing flavonoid concentrations in root exudates enhance associations between arbuscular mycorrhizal fungi and an invasive plant

**DOI:** 10.1038/s41396-021-00894-1

**Published:** 2021-02-10

**Authors:** Baoliang Tian, Yingchun Pei, Wei Huang, Jianqing Ding, Evan Siemann

**Affiliations:** 1 State Key Laboratory of Crop Stress Adaptation and Improvement, School of Life Sciences, Henan University, Kaifeng, Henan China; 2 Key Lab Aquatic Botany & Watershed Ecology, Wuhan Botanical Garden, Chinese Academy of Sciences, Wuhan, Hubei China; 3 Center of Conservation Biology, Core Botanical Gardens, Chinese Academy of Sciences, Wuhan, Hubei China; 4 Biosciences Department, Rice University, Houston, TX USA

**Keywords:** Population dynamics, Community ecology, Plant ecology

## Abstract

Many invasive plants have enhanced mutualistic arbuscular mycorrhizal (AM) fungal associations, however, mechanisms underlying differences in AM fungal associations between introduced and native populations of invasive plants have not been explored. Here we test the hypothesis that variation in root exudate chemicals in invasive populations affects AM fungal colonization and then impacts plant performance. We examined flavonoids (quercetin and quercitrin) in root exudates of native and introduced populations of the invasive plant *Triadica sebifera* and tested their effects on AM fungi and plant performance. We found that plants from introduced populations had higher concentrations of quercetin in root exudates, greater AM fungal colonization and higher biomass. Applying root exudates more strongly increased AM fungal colonization of target plants and AM fungal spore germination when exudate donors were from introduced populations. The role of root exudate chemicals was further confirmed by decreased AM fungal colonization when activated charcoal was added into soil. Moreover, addition of quercetin into soil increased AM fungal colonization, indicating quercetin might be a key chemical signal stimulating AM fungal associations. Together these results suggest genetic differences in root exudate flavonoids play an important role in enhancing AM fungal associations and invasive plants’ performance, thus considering root exudate chemicals is critical to unveiling mechanisms governing shifting plant-soil microbe interactions during plant invasions.

## Introduction

Mutualisms (e.g., mycorrhizae, rhizobia) are key plant biotic interactions with large impacts on plant fitness [1–3]. The strength and direction of these interactions, however, could be altered under global environmental change, such as species invasions, which often create novel biotic interactions [4, 5]. Specifically, enhanced plant-arbuscular mycorrhizal (AM) fungal associations with introduced plants have been implicated in plant invasion success [6, 7]. Although numerous studies have documented the role of such increased mutualism in promoting invasive plant growth [8, 9], mechanisms underlying the differences in AM fungal associations between introduced and native populations of invasive plants are not yet explored. Filling this knowledge gap is critical for unraveling mechanisms underlying evolutionary and ecological linkages in plant–AM fungi interactions and plant-soil feedbacks [10].

Root exudates have been reported to play a crucial role in regulating plant–AM fungi interactions and shaping soil microbial community [11, 12]. Studies show that addition of root exudates can significantly stimulate both AM fungal spore germination rates and hyphal growth [13, 14]. For invasive plants, one study found that, compared to root extracts from plants of native populations of *Solidago canadensis*, root extracts of introduced populations increased their competitive ability while activated carbon eliminated these differences between populations [15, 16]. This suggests that differences in root exudates among populations of invasive plants may play a role in such differences in plant performance. Moreover, adding active carbon that absorbs chemicals to the soil was also found to reduce *S. canadensis* AM fungal colonization and change AM fungal community composition, which strengthens the case that root exudates play a key role in plant-soil microbe interactions [16]. However, to date, there is no direct evidence showing root exudate chemicals differ between native and introduced populations of invasive plants and then affect AM fungal associations.

Secondary chemicals are key root exudate components that could significantly affect AM fungal growth and colonization [17–21]. In particular, flavonoids, such as quercetin and quercitrin, have been reported to stimulate AM fungal spore germination and/or hyphal growth [22, 23]. For example, a study on alfalfa (*Medicago sativa*) showed that quercetin, which is naturally released from alfalfa seeds, increased spore germination, hyphal elongation, and branching of *Glomus* AM fungal species in vitro [22]. Additionally, application of quercetin which occurs in carrot (*Daucus carota*) root exudates stimulated hyphal growth of *Gigaspora margarita* [23]. Moreover, application of flavones can increase AM fungal colonization rate [24]. Therefore, understanding how such secondary chemicals with strong roles in AM fungal colonization change during plant invasion is crucial for providing insights into the mechanisms governing invasive plant–AM fungi interactions.

In this study, our model species Chinese tallow tree (*Triadica sebifera* (L.) Small, hereafter ‘*Triadica*’; synonym *Sapium sebiferum*) was introduced into United States in the 18th century then spread rapidly in the southeastern US [25]. Previous studies show that *Triadica* plants from introduced populations (US) are faster-growing and have lower herbivore resistance than those from native populations (China) perhaps in response to low herbivory in its introduced range [26–28]. Our studies on biogeographical variation in secondary chemicals also show *Triadica* plants from introduced populations have higher flavonoids and lower tannins than plants from native populations [29, 30]. Moreover, plants from introduced populations have higher AM fungal colonization than plants from native populations which may contribute to its invasion success [31, 32].

Here we examined the role of genetic variation in amounts of flavonoids in root exudates of *Triadica* seedlings on AM fungal spore germination, AM fungal colonization, and plant growth in lab, greenhouse and common garden studies in China and US. We tested the specific predictions: (1) root exudate secondary chemicals, such as flavonoids will differ between native and introduced populations, (2) chemicals that have higher concentrations in introduced populations will enhance AM fungal spore germination, (3) the effects of root exudates on AM fungal spore germination and colonization will be stronger for exudates of introduced than native populations, (4) activated carbon will eliminate differences in the effects of introduced vs. native population root exudates. These predictions, however, have never been tested with any invasive plant species.

## Materials and methods

### Seeds collection and germination

We collected *T. sebifera* seeds by hand from populations in both the introduced (US—16 populations in total) and native (China—14 populations in total) ranges (for details see Table S1). At each population, we haphazardly selected 5–10 trees, and harvested thousands of seeds from each tree. In the laboratory, we removed the waxy coats around these seeds by hand after immersing them in a mixture of water and laundry detergent (10 g/L) for 24 h [29]. Then, we rinsed, surface sterilized (10% bleach), and dried them. In order to improve germination, we put these seeds in wet sand and stored them in the refrigerator (4 °C) for at least 30 days. In spring, we sowed these seeds in greenhouse trays (50 holes/tray) which were filled with sterilized (autoclave at 121 °C for 30 min) commercial potting soil, and then kept them in an open-sided greenhouse at Henan University in Kaifeng, Henan, China (34°49′13′′ N, 114°18′18′′ E) or unheated greenhouse at Rice University, Houston, TX USA (29°43′08′′ N 95°24′11′′ W). After seeds germinated and seedlings reached the 4 true leaf stage, we selected similar size seedlings to conduct the following experiments.

### Common garden experiment—differences in AM fungal colonization and plant growth

To investigate the differences in AM fungal colonization and growth between plants from introduced (US) and native populations (CH), we carried out a common garden experiment at Henan University. We collected soil in a corn field, which includes most common AM fungal species based on previous reports [33, 34]. It was a sandy soil with total nitrogen and total phosphorus of 1.9 g/kg (DW) and 0.6 g/kg (DW), respectively, and pH of 7.68. We removed surface litter before collecting topsoil (10–15 cm depth) and then combined equal parts of soil and fine sand in 132 pots (21 cm × 16 cm, ~3 kg of soil mix each) after they were passed through a 1-cm mesh screen. We planted seedlings from 22 populations (12 native and 10 introduced populations, 6 seedlings of each population, Table S1) individually in these prepared pots and placed them in the open-sided greenhouse. We protected them from herbivores with nylon mesh (16 openings/cm) cages during the experiment. After 60 and 90 days, we harvested 3 seedlings from each population as 3 reps each time and carefully washed their whole roots from the soil. We collected ~30 fresh fine roots (>1 cm/segment) from each plant root to measure AM fungal colonization. In brief, we cleared (in 10% KOH), bleached, acidified, and stained (trypan blue) these samples then slide mounted 30 one cm long pieces of fine root for each plant [7]. AM fungal colonization was determined by the gridline intersect method with 300 intersection points per plant [35]. We dried and weighed the roots and shoots.

### Collection of root exudates and flavonoids analysis for root exudates

Our previous study found higher concentrations of flavonoids but lower concentrations of tannins in roots of introduced populations of *T. sebifera* than in native populations [17] with quercetin and quercitrin being the main flavonoids [28, 30]. In our pilot experiment, we only detected quercetin and quercitrin in root exudates but no other flavonoids. Therefore, in this study we focused on quercetin and quercitrin in root exudates and their functions. We determined their amounts in root exudates from native (China) and introduced (US) populations at Henan University. We filled 132 glass beakers (1000 ml) with Hoagland’s solution [36] and covered the opening with a foam board with a hole in its center. We took 6 seedlings from each of 22 populations (12 native, 10 introduced, Table S1) and carefully washed the soil from their roots with tap water, then transplanted them individually into the beakers (1 seedling per beaker) and fixed them with a sponge. Because of mortality, only 80 plants of 17 populations (9 native, 8 introduced) survived until exudate collection. The odds of a plant dying did not depend on population origin (*F*_1,20_ = 3.7, *P* = 0.0679) or population (*Z* = 1.3, *P* = 0.0937). We checked these glass beakers and filled them with Hoagland’s solution every day.

After these plants grew for 57 or 87 days in an open-sided greenhouse with a typical temperature range of 18 °C (night) to 28 °C (day) and 13–14 h of natural daylight, we put DI water into these beakers instead of Hoagland’s solution to minimize the effects of environments on root exudates. Three days later (i.e., at 60 and 90 days) these plants were harvested to obtain their dry root mass. The root exudates were dried at 40 °C under vacuum by rotary evaporators. Then we extracted the chemicals from these concentrates in 3 ml of methanol solution with 0.4% phosphoric acid water (48:52, v:v) and filtered them through 0.22 μm hydrophobic membranes. The concentrations of quercetin and quercitrin were assessed by high-performance liquid chromatography [30]. In brief, 20 μl of extract was injected into an HPLC with a ZORBAX Eclipse C18 column (4.6 × 250 mm, 5 μm; Agilent, Santa Clara, CA, USA) with the following flow: 1.0 mL min^−1^ with a 100% methanol (B) and 0.4% phosphoric acid in water (A) as the mobile phase. The gradient was as follows: 0–10 min 52:48 (A:B); 10–24 min 48:52 (A:B). UV absorbance spectra were recorded at 254 nm. The concentrations of flavonoid compounds were calculated and standardized by peak areas of standards of known concentrations.

### Root exudate addition experiment—effects of different populations on AM fungal colonization

In order to investigate the role that root exudates play in the interactions between AM fungi and plants, we conducted an experiment in which exudates were collected from plants in liquid (donor) and applied to the soils of other plants (target). The exudate donor plants were grown in 1080 (two venues: 540 seedlings at Rice University and 540 seedlings at Henan University) containers, each with 1000 ml of Hoagland’s solution, that each had a foam board top with a hole and a bottom drain tube that could be regulated. At each venue, we washed the soil from ~500 sets of plants (US = 465, China = 504) from native (8 populations for venue US and 7 populations for venue CH) or introduced (13 populations for venue US and 12 populations for venue CH) populations and secured them (3 plants per container) in the containers using sponges (details in Table S1). The remaining containers were left as plant-free controls. We started the application experiment after 7 days.

For exudate target plants, we collected the soil from different sites in the introduced or native ranges (See Table S1). At each site, we collected soil under the canopy of a *T. sebifera* tree (Home soil) and that more than 3 meters away from the canopy of a *T. sebifera* tree (Away soil). We collected the topsoil to a depth of 15 cm after removing the surface litter, air dried them, and screened them (1 cm mesh). These soils were mixed with vermiculite (1:2 volume). Then we used these mixes to fill 1080 pots (15 cm × 12 cm; 540/venue). Each pot in China received a mixed soil from a site in China and each pot in US received soil from a single small area within a site in the US. We transplanted a seedling from a native (12 populations for venue US and 3 populations for venue CH, See Table S1) or introduced (13 populations for venue US and 5 populations for venue CH, See the Table S1) population into each pot (270 of each per venue). We randomly assigned a target plant to each set of donor plants or water only controls.

Every 4 days we changed the Hoagland’s solution to DI water for 3 days to collect root exudates from donor plants. Then we applied this water solution from a donor set to its target plant. After 70 days, we harvested the target seedlings, kept a fine root sample for AM fungal colonization determination, then dried and weighed leaves, stems, and roots.

### Chemical addition experiment—quercetin and quercitrin effects on AM fungal colonization

We transplanted 391 seedlings from 8 native populations (CH) and 9 introduced populations (US) into 391 pots with field soil (1.3 kg/pot) in nylon mesh cages at Henan University. To test the effect of quercetin and quercitrin on AM fungal colonization, we prepared solution of quercetin or quercitrin in acetone (10 mg/mL) (acetone did not affect AM fungal colonization based on our preliminary experiment). Then these solutions were diluted in water to 2 concentrations (1 mg/L and 10 mg/L) based on the result of chemical analyses of root exudates and the 0.1% of acetone in water as control. We watered 15 ml of solution (5 reps per population) or water (3 reps per population) around the base of seedling stems every 3 days (16 times in total). Four plants died (3 in quercitrin application treatment, 1 in quercetin application treatment). After 70 days, we collected seedlings by cutting at ground level and collected fine roots to test AM fungal colonization.

### Activated carbon experiment—AM fungal colonization with inactivated chemicals

In order to verify the chemicals in root exudates play a key role in the relationship between AM fungi and plant roots, we conducted an experiment at Henan University with activated carbon (AC) addition to block bioactivity of root exudate chemicals. We filled plastic pots in mesh cages at Henan University with either 1.3 kg of field soil (*N* = 78) or field soils amended with activated carbon (*N* = 78, Sinopharm Chemical Reagent Co., Ltd, Beijing, China) added as 1:500 v:v. We transplanted seedlings from 13 populations (6 native and 7 introduced, Table S1) into the pots with 6 seedlings for each population. Eighteen seedlings died during this experiment (12 seedlings from AC treatment, 6 seedlings from control). After 70 days, we harvested plants and used a fine root sample to determine AM fungal colonization.

### Field survey of AM fungal assemblages

We collected rhizosphere soil from 3 sites in China (Dawu, Hubei, 31°28′N, 114°16′E; Wuhan, Hubei, 30°32′N, 114°25′E; Guilin, Guangxi, 25°04′N, 110°18′E) for AM fungal species identification via high-throughput sequencing. At each of these sites, we selected 3 *T. sebifera* trees per site and dug the soil close to the tree trunk until its root branch was found. We collected soils from 3 roots per plant. We removed the bulk soil from these roots by shaking, and then collected the soil remaining on these roots using brushes (1 new brush per tree). The rhizosphere soils on the roots from same tree were mixed fully. About 5 g of fresh rhizosphere soil from one tree was collected and stored in dry ice and ultra-low temperature freezer (−80 °C) until they were used to test the AM fungi abundance based on high-throughput sequencing [37, 38].

For DNA extraction, microbial DNA was extracted from the prepared samples (0.25 g soil per sample) using the E.Z.N.A.® soil DNA Kit (Omega Bio-tek, Norcross, GA, U.S.) according to the manufacturer’s protocols. The DNA concentration and purification were determined by NanoDrop 2000 UV-vis spectrophotometer (Thermo Scientific, Wilmington, USA), and DNA quality was checked by 1% agarose gel electrophoresis [39].

For the PCR amplification, nested PCR was conducted to amplify the AM fungi 18S rRNA. The primer pairs AML1 (5′-ATCAACTTTCGATGGTAGGATAGA-3′) and AML1 (5′-GAACCCAAACACTTTGGTTTCC-3′) were used in the first run. The primer pairs AMV4.5NF (5′-AAGCTCGTAGTTGAATTTCG-3′) and AMDGR (5′-CCCAACTATCCCTATTAATCAT-3′) were used in the second run in the thermocycler PCR system (GeneAmp 9700, ABI, USA). The PCR reactions were conducted using the program according to Xiao et al. [39].

For each sample, purified amplicons were pooled in equimolar and paired-end sequenced (2 × 300) on an Illumina MiSeq platform (Illumina, San Diego, USA) according to the standard protocols of Majorbio Bio-Pharm Technology Co. Ltd. (Shanghai, China). The raw fastq files were quality-filtered by Trimmomatic and merged by FLASH with the following criteria: (i) the reads were truncated at any site receiving an average quality score <20 over a 50 bp sliding window. (ii) Sequences whose overlap being longer than 10 bp were merged according to their overlap with mismatch no more than 2 bp. (iii) Sequences of each sample were separated according to barcodes (exactly matching) and Primers (allowing 2 nucleotide mismatching), and reads containing ambiguous bases were removed. Operational taxonomic units (OTUs) were clustered with 97% similarity cutoff using UPARSE (version 7.1 http://drive5.com/uparse/) with a novel “greedy” algorithm that performs chimera filtering and out clustering simultaneously. The taxonomy of each 16S rRNA gene sequence was analyzed by RDP Classifier algorithm (http://rdp.cme.msu.edu/) against the Silva (SSU123) 16S rRNA database using a confidence threshold of 70%. Finally, we estimated the abundances of genera as the read numbers of OTUs.

### Spore germination experiment—effects of exudates on germination

We examined the effect of crude root exudates on AM fungal spore germination by the paper disk diffusion method according to Akiyama [40]. Because the field survey indicated that *Glomus* was dominant and *Glomus mosseae* has been reported as one the most common species [40, 41], we tested this species in our study. We obtained the strain from Bank of Glomales in China (BGC), Institute of Plant Nutrition and Resources, Beijing Academy of Agriculture and Forestry Sciences. We propagated *G. mosseae* AM fungal spores according to published methods [42]._._ We collected crude root exudates from seedlings which were planted in soil or Hoagland’s solution. For the seedlings planted in soil, we selected 75 similar size seedlings from 15 populations (9 CH and 6 US). After they grew for 27, 57, or 87 days, we washed them from soil and transferred them individually into 1000 ml of DI water. We collected root exudates after 3 days. For seedlings planted in liquid, we selected 30 of similar size from 10 populations (4 CH and 6 US) washed soil from their roots and put them individually into beakers with 1000 ml of Hoagland’s solution. We pumped air into the solutions with air pump every day to supply enough oxygen to roots and added new Hoagland’s solution every day to provide them enough nutrition. After 87 days of growth, we replaced the solution with DI water for 3 days to collect their root exudates.

To test the spore germination, we first put a paper disk (6 mm in diameter), which was soaked with 20 μl of crude exudate or water, on the center of a dish that contained 0.2% Phytagel and 3 mM MgSO_4_. We inoculated ten *G. mosseae* spores in the dish (spaced 15 mm apart). The control was 20 μl of water. Each dish was treated as a replicate, with five replicates per treatment (control and root exudates from every seedling). We placed dishes in an incubator at 28 °C and recorded the total number of germinated spores in each dish after 5, 10, 15, and 20 days.

### Spore germination experiment—effects of quercetin and quercitrin on germination

In order to evaluate the bioactivity of quercetin and quercitrin on AM fungi, we tested the effect of different doses of the two chemicals on spore germination. In this experiment, the tested doses of quercetin or quercitrin were 0.02, 0.2, and 2 mg in 20 ml of culture medium in a petri dish after the two chemicals were dissolved in 1 ml of acetone. The culture medium included 0.2% Phytagel and 3 mM MgSO_4_. We mixed quercetin or quercitrin in culture medium before it became solid (~35 °C). After 12 h, we inoculated ten *G. mosseae* spores in each petri dish and cultured them at 28 °C in an incubator [43]. The positive control and negative control were 1 ml of water and acetone, respectively. There were five replicates (i.e., dishes) per treatment. We recorded the total number of germinated spores in each dish after 5, 10, 15, and 20 days.

### Statistical analyses

We performed mixed model ANOVAs (proc mixed) to examine the dependence of AM fungal colonization or plant mass on the fixed effects population origin, plant age (60 or 90 days), and their interaction and the random effect population (origin) in the common garden experiment. We used partial difference contrast tests to distinguish among means for significant fixed effect factors with more than two levels in this and other ANOVAs.

We used a mixed model ANOVA with the fixed effects plant population origin (US vs. China), plant age (60 or 90 days), and their interactions and the random effect plant population nested in origin to determine their effects on the root mass specific concentrations of flavonoids (quercetin or quercitrin) in root exudates in the root exudate production experiment. These data were log transformed to meet assumptions of ANOVA.

For the exudate application experiment, we used mixed model ANOVAs to test the dependence of mycorrhizal colonization or plant mass on the fixed effects population origin of target recipient plant (“T”, China or US), venue (“V”, China or US experimental block), population origin of exudate donor plant (“E”, China or US), home vs. away soil collection (“H”), and their interactions and the random effects target population nested in target origin, exudate population nested in exudate origin, and soil site (“S”) nested in venue. We fit a family of models that included all combinations of the factors T, V, E, and H. For each set of factors, we first fit full-interaction models then all lower level interaction levels (to main effects only). We used AIC values to select the best fitting models for mycorrhizal colonization and plant mass. Model selection indicated the best fit model for mycorrhizal colonization was the full-interaction model with all factors (Table S2). For mass, the best fitting model was one with only target origin, venue, and their interaction as fixed effects (Table S2).

For the chemical addition experiment, we fit mixed model ANOVAs to test the dependence of mycorrhizal colonization on the fixed effects population origin of target recipient plant (China or US), chemical addition (control, quercetin, quercitrin), concentration nested in chemical addition (0, 0.24 or 2.4 mg), their interaction and the random effect population (origin).

For the activated carbon experiment, we fit a mixed model ANOVA to test the dependence of mycorrhizal colonization on the fixed effects population origin of target recipient plant (China or US), charcoal addition (control or addition), their interaction and the random effect population (origin).

For the exudate and spore germination experiment, we used an ANOVA to test the dependence of AM fungal spore germination odds (ln[number of germinating/number of not germinating]) on root exudate plant origin (US or China), growth medium (soil or liquid), plant age (30, 60, 90 days), days after start of germination (5, 10, 15, 20 days) and their interactions as fixed effects along with the random term population nested in origin along with terms to account for the non-independence from exudates from a single plant being applied to multiple petri dishes and germination being measured repeatedly on the same petri dish.

For the chemical and spore germination experiment, we used ANOVA to test the effect of different concentrations of quercetin and quercitrin along with water or acetone controls (“treatment”) on AM fungal spore germination in two experimental blocks over 4 time periods (categorical variable: 5, 10, 15, or 20 days).

## Results

### Common garden experiment—plant growth, AM fungi, and flavonoids in root exudates

**Fig. 1:**
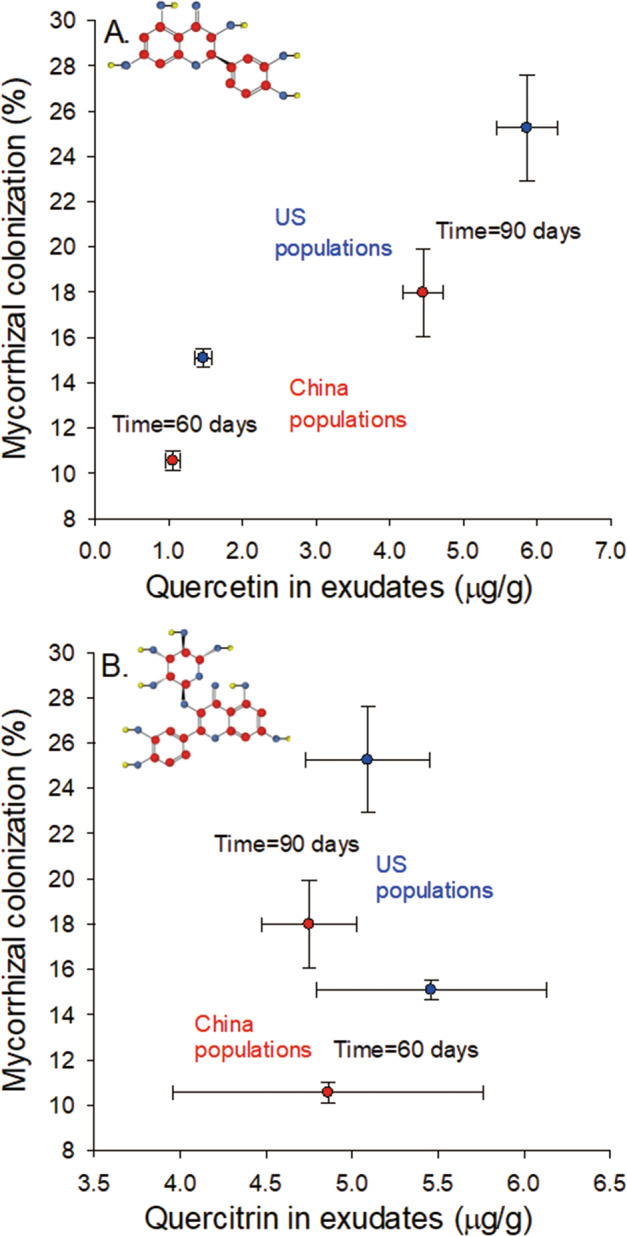
The relationships between AM fungal colonization and the root mass specific concentrations of flavonoids in the root exudates of plants from native (China, red symbols) and introduced (US, blue symbols) populations at 60 and 90 days. **A** quercetin. **B** quercitrin. Means ± SE.

Plants from introduced populations had significantly higher AM fungal colonization than native population plants at both 60 and 90 days (Table 1, Fig. 1A, B). Introduced and native populations had similar masses at 60 days but plants from introduced populations were significantly larger than those from native populations at 90 days (Table 1; native populations (China) = 4.36 ± 0.25 g, introduced populations (US) = 5.31 ± 0.16 g).

**Table 1:** The dependence of AM fungal colonization and plant mass on population origin (China or US), time (categorical variable: 60 or 90 days), and their interaction as fixed effects and population nested in origin as a random effect in the common garden experiment.

		AM fungi	Mass
Fixed effects	DF	*P*-value	*P*-value
Origin	1,20	**0.0044**	**0.0139**
Time	1,108	**<0.0001**	**<0.0001**
Origin*time	1,108	0.3553	0.1647
**Random effect**		***P*-value**	***P*-value**
Population (origin)		0.1386	**0.0327**

Significant results are indicated in bold.

Population origin significantly affected the root mass specific concentrations of quercetin in root exudates, with plants of introduced populations having higher concentrations than the native ones (Table 2, Fig. 1A). Older plants (90 days) had higher root mass specific concentrations of quercetin. The root mass specific concentrations of quercitrin did not depend significantly on population origin or plant age (Table 2, Fig. 1B).

**Table 2:** The dependence of root mass specific flavonoid concentrations (quercetin or quercitrin) in the root exudate production experiment on population origin (China or US), time (categorical variable: 60 or 90 days), and their interactions as fixed effects and population nested in origin as a random effect.

		Quercetin	Quercitrin
Fixed effects	DF	*P*-value	*P*-value
Origin	1,15	**0.0019**	0.5008
Time	1,61	**<0.0001**	0.6660
Origin*time	1,61	0.5337	0.8034
**Random effect**		***P*-value**	***P*-value**
Population (origin)		0.3080	0.5711

Significant results are indicated in bold.

### Root exudate addition experiment—effects on AM fungal colonization

**Fig. 2:**
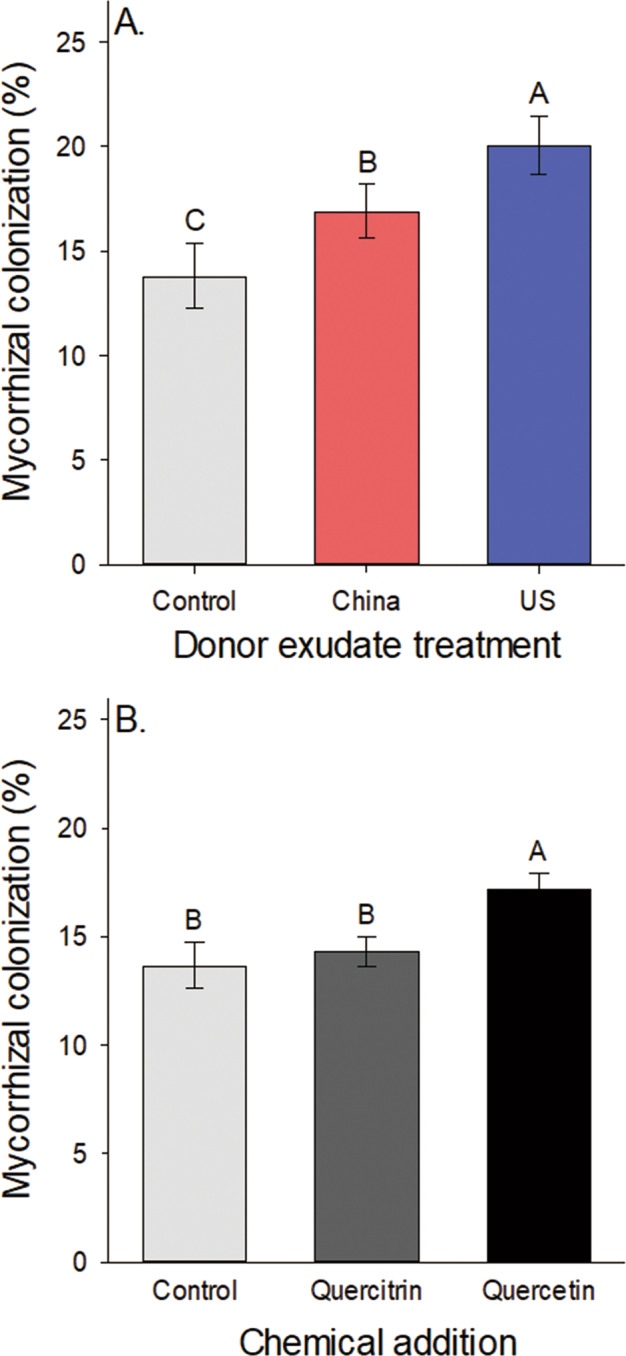
The dependence of mycorrhizal colonization on root exudate and chemical addition. **A** AM fungal colonization of target seedlings when they received root exudates from donor plants of different population origins (native: China and introduced: US) in the exudate addition experiment. **B** AM fungal colonization of seedlings that received quercetin or quercitrin. Means ± SE. Treatment means with the same letter were not significantly different in post hoc tests (*P* > 0.05).

Mycorrhizal colonization was higher for plants that received exudates compared to controls (only adding water). Adding exudates from plants of introduced populations showed stronger effects on AM fungal growth than those from the plants of native populations (Table 3, Fig. 2A). Experimental venue affected AM fungal growth, with plants grown in China having higher levels of mycorrhizal colonization than those grown in the US (Table 3; China experimental block = 20.66 ± 2.34%, US experimental block = 13.34 ± 1.31%). Mass of target plants depended on the interaction of target plant origin and venue with plants larger in the US venue (but no difference between US and China origin plants) and US origin plants larger than China origin plants in China (Table 3).

**Table 3:** The dependence of mycorrhizal colonization in the exudate application experiment on the fixed effects population origin of target recipient plant (“T”, China or US), venue (“V”, China or US experimental block), population origin of exudate donor plant (“E”, China or US), home vs. away soil collection (“H”), and their interactions and the random effects target population nested in target origin, exudate population nested in exudate origin, and soil site (“S”) nested in venue.

	AM fungi		Mass	
Fixed effects	DF	*P*	DF	*P*
T	1,25	0.2388	1,25	**0.0202**
V	1,7	**0.0217**	1,7	**0.0024**
T × V	1,984	0.4140	1,1028	**0.0158**
E	2,24	**0.0001**		
T × E	2,984	0.1841		
V × E	2,984	0.3535		
T × V × E	2,984	0.1652		
H	1,984	0.5256		
V × H	1,984	0.4164		
E × H	2,984	0.2283		
T × H	1,984	0.3505		
T × E × H	2,984	0.8954		
V × E × H	2,984	0.4307		
T × V × H	1,984	0.2233		
T × V × E × H	2,984	0.1041		
**Random effects**	**DF**	***P***	**DF**	***P***
TP (T)	25	0.1498	25	**0.0015**
S (V)	**7**	**0.0470**	7	**0.0336**
EP (E)	24	0.9021		

Model selection indicated the best fit model for mass was one with only target origin, venue and their interaction as fixed effects (see Table S1 for model selection results). Significant results are indicated in bold.

### Chemical addition experiment—quercetin and quercitrin effects on AM fungal colonization

In the chemical addition experiment, plants that received quercetin had higher AM fungal colonization than those in control or quercitrin addition treatments (Table S3, Fig. 2B) and colonization levels were higher for plants from introduced populations than those from native populations [Table S3; Native populations (China) = 14.34 ± 0.74%, Introduced populations (US) = 17.67 ± 0.77%].

### Activated carbon experiment—effects on AM fungal colonization

**Fig. 3:**
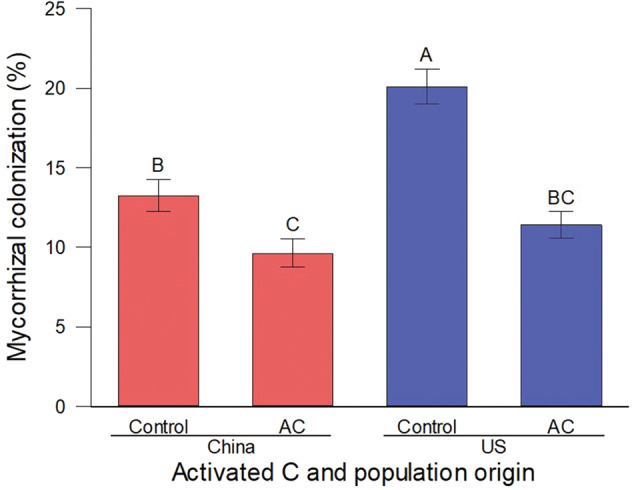
The dependence of AM fungal colonization on population origin and activated charcoal addition. Means ± SE. Treatment means with the same letter were not significantly different in post hoc tests (*P* > 0.05).

Adding activated carbon (Charcoal) significantly decreased AM fungal colonization for plants from both introduced and native populations. This effect was stronger for plants from introduced populations than those from native populations, as indicated by the interactive effect of treatment and origin (Table S4, Fig. 3).

### Dominant genus of AM fungi around *T. sebifera* trees and spore germination experiments

**Fig. 4:**
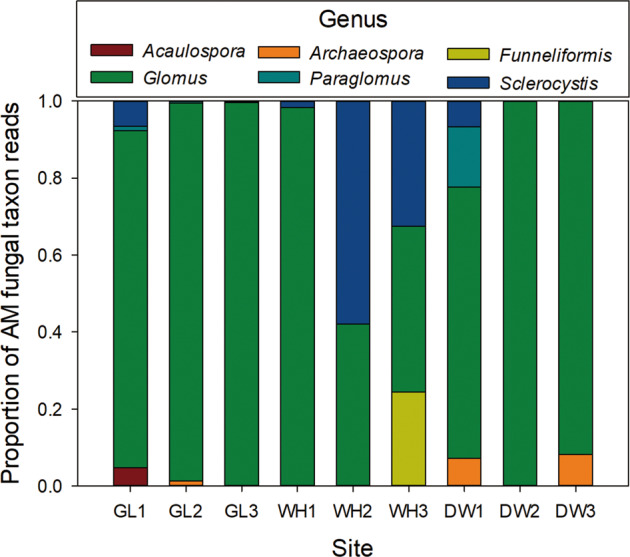
The abundance of AM fungal genera in the rhizosphere soil of *T. sebifera*. The proportion of AM fungal taxa reads from different genera in rhizosphere soil of *T. sebifera* in three samples at each of 3 sites: Guilin, Guangxi (GL); Wuhan, Hubei (WH); Dawu, Hubei (DW). Taxon bar height (“Proportions” on the *y*-axis) indicate the relative ratio (%) of taxonomic groups in each soil sample. *Glomus* (green bars) was the most abundant in all the soil samples.

Based on the high-throughput sequencing test, *Glomus spp*. were found to be the most abundant (i.e., largest number of reads) in the rhizosphere soil from *T. sebifera* trees (Fig. 4).

**Fig. 5:**
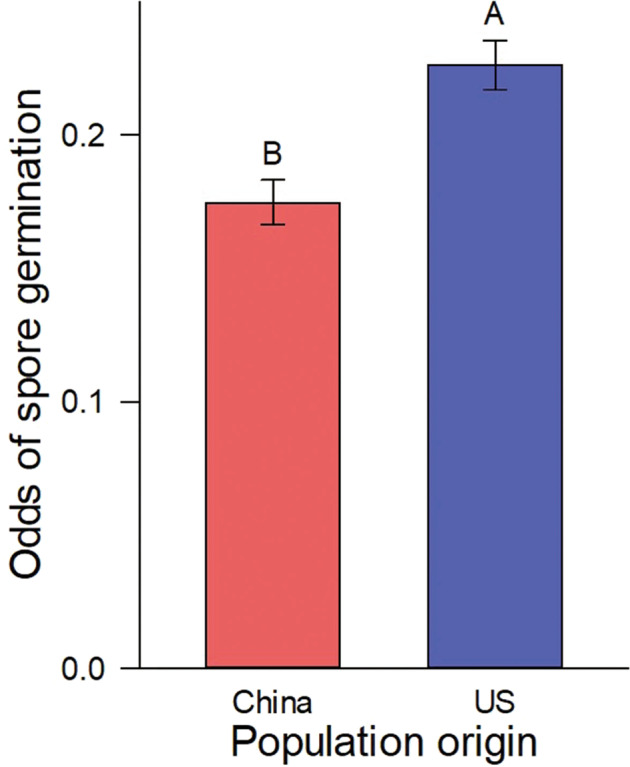
The effect of root exudates on AM fungal spore germination. The dependence of the odds of *Glomus mosseae* spore germination on the population origin of *T. sebifera* plant root exudates (11 native populations and 12 introduced populations). Means ± SE. Treatment means with the same letter were not significantly different in post hoc tests (*P* > 0.05).

Root exudate population origin significantly affected *Glomus* spore germination. Spores that received exudates from plants of introduced populations had higher germination than spores that received exudates from plants of native populations (Table S5, Fig. 5). Germination rates of spores that received exudates from plants grown in soil were on average similar with those from plants grown in liquid at the same days (Fig. S1).

Spore germination was higher with intermediate quercetin or low or, and intermediate quercitrin addition at the dose of 0.2 mg compared to water or acetone treatments (Table S6, Fig. S2).

## Discussion

In this study we show clearly that *Triadica* plants from introduced populations had higher amounts of quercetin in their root exudates than those from native populations. Our exudate and flavonoids addition experiments, as well as the active carbon experiments, further reveal the role of this flavonoid in enhancing the AM fungal associations of introduced populations. To our knowledge, our work is the first to report the variation of root exudate chemicals in native and introduced populations of invasive plants and document the effects of such differences in root exudate chemicals in shaping AM fungal associations. This may provide new insights into how plant-soil feedbacks are mediated by root exudate chemicals and the mechanisms governing shifting plant-soil microbe interactions during plant invasions.

Previous studies have reported that plant’s root exudates could facilitate AM fungal colonization of roots [44, 45]. Furthermore, some kinds of flavonoids have been reported to have bioactivity on AM fungal spore germination [46] and hyphal growth [47, 48], and then play a role in fungal invasion and arbuscule formation inside the root [49]. Consistently, our experiments on AM fungi and plant growth found positive effects of adding root exudates and quercetin in greenhouse experiments and strong bioactivity of quercetin on AM fungal spore germination. Our analyses of root exudates did not allow us to distinguish between greater concentrations of quercetin in exudates of plants from introduced populations or greater quantities of exudates. Moreover, the similar AM fungal colonization between plants from native and introduced populations when they were inoculated with root exudates from plants from introduced populations strongly supports a role for exudates. Application of activated carbon could change physical and chemical properties of the soil, and might eliminate effects of all chemical signals, thus the stronger negative effects of adding active carbon on the AM fungi of the introduced populations also indicated the greater activity of chemicals in their root exudates.

Our lab bioassay with root exudates and AM fungal spore germination further confirmed such stronger effects by root exudates of plants from the introduced populations. These stronger effects of the US exudates on AM fungal colonization may be, at least partly, attributed to the increasing flavonoid effects in introduced populations. Although flavonoids have long been known to enhance AM fungal growth in many plants [46, 49, 50], our study is the first to find such increasing flavonoids increased AM fungal colonization in invasive plants. We acknowledge that there may be also some other chemicals in root exudates affecting AM fungal colonization such as fatty acids [51], isoflavonoids [52], and strigolactone [53, 54]. Thus, our experiment with application of activated carbon might also eliminate effects of these chemicals and their variations in root exudates between plants of native and introduced populations should also be examined in the future. In addition, although we found compelling results for the spore germination of the most abundant AM fungal group in our field survey, the effects of root exudates may vary among taxa. Indeed, the effect of activated carbon addition that presumably bound root exudate chemicals on *S. canadensis* mass varied with the species of AM fungal inoculum suggesting that AM fungal taxa vary in their responses to root exudates [16]. Further, establishment of AM fungal associations involves steps other than spore germination that may be sensitive to root exudate chemicals (e.g., hyphal elongation, chemotaxis) that we did not examine in our lab assays. Lastly, in this study, we performed hydroponic experiments to examine the flavonoids in root exudates, thus the detected concentrations of quercetin and quercitrin might differ from those in the rhizosphere soil. Because different concentrations of quercetin and quercitrin could have different effects on AM fungi or plant growth, future studies that directly detect and monitor their content in soil may assist to match chemical concentrations in experiments with real concentrations in the field. This will enable researchers to better establish the relationships between AM fungal colonization and flavonoids under natural conditions, although the outcome may be determined by plant growth stage, soil abiotic and biotic environments and technologies for detecting chemicals. Nevertheless, taken together, these results suggest differences in the release of flavonoids by roots between introduced and native populations of the invasive plant likely influence variations of AM fungal association and plant growth during the plant invasion.

There are several reasons why root exudate flavonoids and their effects on AM fungi may be enhanced in *Triadica* plants of the introduced populations. First, one of the main roles of secondary metabolites is defense against herbivores [55–57]. Increase in flavonoids, including those in root exudates, could reflect broader changes in secondary metabolism in the introduced range where herbivores are rare [58–60], such as a reduction in tannins [30, 61]. Second, flavonoids have also been found to stimulate pathogenic fungal spore germination and to attract hyphal colonization [44, 62]. Thus, releasing flavonoids from the root could potentially increase both AM fungal associations and the risk of infection by soil fungal pathogens. In the native range where soil pathogens are common, the costs of increased root flavonoid exudation are likely larger than in the introduced range where soil fungal pathogens are rare and so there may be directional selection on increased root flavonoid exudation. Third, flavonoids have many functions other than AM fungi stimulation including UV and drought tolerance [63, 64], thus the introduced populations of invasive plants may produce more flavonoids to adapt to novel abiotic environments. For example, a recent study found leaf flavonoids contents in *Triadica* introduced ranges were closely related with UV and solar radiation [65]. In this scenario, increased release of flavonoids by roots reflects generally higher levels of flavonoids produced for other functions. Because a number of biotic and abiotic factors could affect root exudate chemicals, future work is needed to further reveal mechanisms underlying changes in root exudate chemicals in different plant populations [64].

In addition to flavonoids, there are some other environmental factors affecting AM fungal mutualisms, such as soil nutrients and soil microbes [66, 67]. In this study we performed some of our root exudates experiments in both the native and introduced ranges (China vs. US), with variable local soils. The overall difference of AM fungal colonization between ranges might reflect variation of the original abundance of AM fungi, soil P contents or other soil microbes [67–69]. Nevertheless, the patterns of differences between plant origin are consistent: experiments from both countries showing stronger effects of the exudates from plants from introduced populations on AM fungal colonization.

Our findings have important implications for plant invasion biology and microbial ecology by linking root exudation, AM fungal colonization and plant performance. Although increased AM fungal colonization has been reported in many invasive plants (relative to their native neighbors) [70] or introduced populations (relative to native populations of the same species) [8, 31, 32], the mechanisms underlying such differences in AM fungal associations are unclear. Our study clearly shows higher flavonoid concentrations in root exudates increase AM fungal spore germination and AM fungal associations and in turn promote plant performance in *Triadica* introduced populations. Furthermore, differences in root exudate chemical concentrations and AM fungal associations may also impact soil microbes and other soil processes, likely affecting the invasive plant and its neighbors in the community. Thus, changes in root exudate chemicals could promote plant invasions by enhancing plant–AM fungal associations and subsequently impact the ecological effects of invasive plants.

In conclusion we found significant biogeographical differences in a root exudate secondary chemical and its effect on AM fungal spore germination and colonization in an invasive plant. Demonstration of the effects of root exudate chemicals is critical to unveil mechanisms governing shifting plant-soil microbe interactions, such as plant-soil feedbacks during plant invasions. Additionally, together with our previous studies, which show that *Triadica* plants from introduced populations (US) experience less herbivory [26–28] and have higher flavonoids [29, 30] compared with those from native populations, there is the possibility of important interactions between different types of biotic interactions in shaping plant secondary metabolism. Furthermore, as a growing body of literature reports the role of secondary chemicals in plant invasions, we expect chemical-induced shifts in plant-microbial interactions, such as we report here will also apply to other plant invasions. In this context, more studies on root exudate chemicals and their dynamics during invasions are needed to explicitly document their roles in shaping plant-soil microbe interactions.

## Supplementary information

Manuscript data and codes

Supplementary figures, tables, and the link for database
