# Supplementary material for: Increasing flavonoid concentrations in root exudates enhance associations between arbuscular mycorrhizal fungi and an invasive plant
Source: ISME J. 2021 Feb 10;15(7):1919–30. doi: 10.1038/s41396-021-00894-1 (PMC8245413; doi:10.1038/s41396-021-00894-1)
Supplement: Supplementary file 2 — Supplementary figures, tables, and the link for database [file 41396_2021_894_MOESM2_ESM.docx]

**SUPPLEMENTARY INFORMATION**

**Supplement for figure Legends**

**Fig. S1** The odds of spores germinating in dishes treated with exudates from plants from native (China) or introduced (US) populations grown in soil for 30, 60 or 90 days or grown in liquid for 90 days. Means± SE. Treatment means with the same letter were not significantly different in post hoc tests (P>0.05).

**Fig. S2** The odds of spores germinating in dishes treated with different chemicals. CK = water control, acetone = acetone control. Means± SE. Treatment means with the same letter were not significantly different in post hoc tests (P>0.05).

**Supplement for table Legends**

**Table S1:** Populations of *T. sebifera* from the native and introduced ranges used in this study, the years in which seeds were collected (in November), and the experiments in which the seeds were used (CG=common garden, RE=root exudate production, SG=spore germination, E=exudate application, C+=chemical addition, AC=activated carbon). Sites indicated by soil collection were used for target plants in the exudate experiment.

**Table S2.** Results of model selection for analysis of target plant mycorrhizal colonization and mass in the exudate application experiment. AIC values are for models that potentially included the fixed effects population origin of target recipient plant (“T”, China or US), venue (“V”, China or US experimental block), population origin of exudate donor plant (“E”, China or US), home vs. away soil collection (“H”), and their interactions and the random effects target population nested in target origin, exudate population nested in exudate origin, and soil site (“S”) nested in venue. The models with the best fits (lowest AIC) are indicated in underlined, bold font. Model fit (AIC) for full models without the sqrt transformations were much worse (mycorrhizae=8313.0; mass=3192.1).

**Table S3** The dependence of mycorrhizal colonization on the fixed effects population origin of target recipient plant (China or US), chemical addition (control, quercetin, quercitrin), concentration nested in chemical addition (0, 0.24 or 2.4 mg in total), their interaction and the random effect population (origin) in the chemical addition experiment. Significant results are shown in bold.

**Table S4** The dependence of mycorrhizal colonization on the fixed effects population origin of target recipient plant (China or US), charcoal addition (control or addition), their interaction and the random effect population (origin) in the activated carbon experiment. Significant results shown in bold.

**Table S5** The dependence of AM fungal spore germination odds on root exudate plant origin (US or China), growth medium (soil or liquid), plant age (30, 60, 90 days), days after start of germination (5, 10, 15, 20 days) and their interactions as fixed effects along with the random term population nested in origin along with terms to account for the non-independence from exudates from a single plant being applied to multiple petri dishes and germination being measured repeatedly on the same petri dish.

**Table S6** The effect of different concentrations of quercetin and quercitrin along with water or acetone controls (“treatment”) on AM fungal spore germination in two experimental blocks over 4 time periods (categorical variable: 5, 10, 15, or 20 days). Significant results are indicated in bold.

**The public database for article data and proc codes in SAS:** [**https://datadryad.org/stash/share/e8xNu0H14bl9r1t9TJ5XermVJpdljN43F2cno8H6oK0**](https://datadryad.org/stash/share/e8xNu0H14bl9r1t9TJ5XermVJpdljN43F2cno8H6oK0)

**Supplement for figures**

**Fig. S1**

**
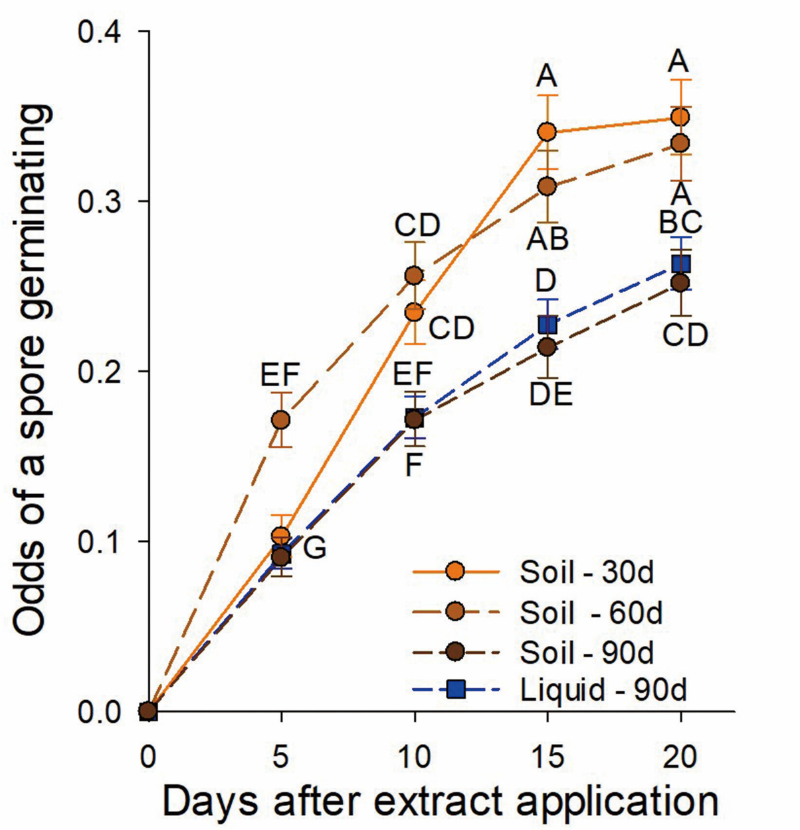
**

**Fig S2**

**
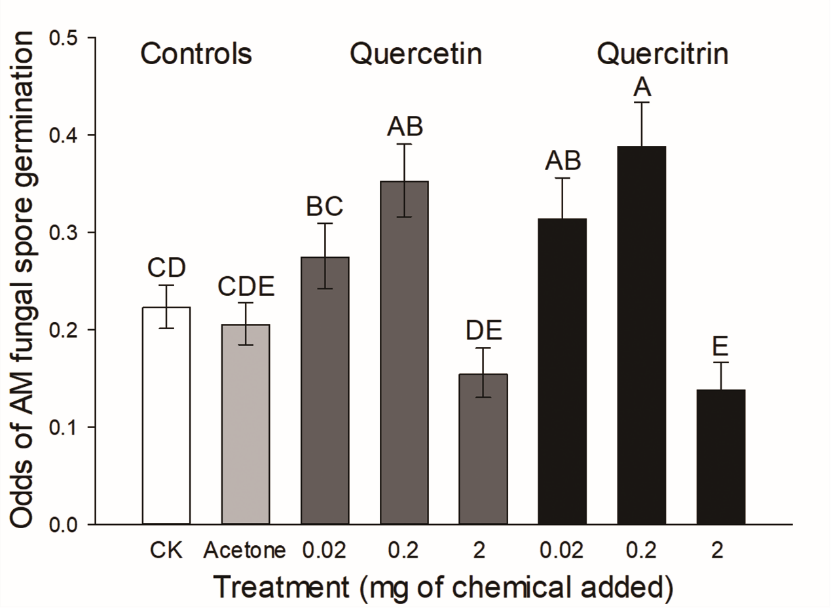
**

**Supplement for tables**

**Table S1:**

|  |  |  |  |  | | | |  |  | | | | | |  |  |
| --- | --- | --- | --- | --- | --- | --- | --- | --- | --- | --- | --- | --- | --- | --- | --- | --- |
| **ID** | **Location** | **Latitude** | **Longitude** | **Seed collection year** | | | |  | **Experiment (seeds)** | | | | | |  | **Soil collection** |
|  |  |  |  | **2015** | **2016** | **2017** | **2018** |  | **CG** | **RE** | **SG** | **E** | **C+** | **AC** |  | **E** |
| Native populations (in China) | | | |  |  |  |  |  |  |  |  |  |  |  |  |  |
| CH-DW | Dawu, Hubei | 31°28′N | 114°16'E | x | x | x | x |  | x | x | x | x |  | x |  |  |
| CH-GL | Guilin, Guangxi | 25°04′N | 110°18′E | x | x | x | x |  | x | x | x | x | x | x |  |  |
| CH-HeS | Hengshan, Hunan | 27°14′N | 112°46′E | x | x | x | x |  | x | x | x | x | x |  |  |  |
| CH-HF | Hefei, Anhui | 31°50′N | 117°09′E | x | x | x | x |  | x | x | x | x | x | x |  |  |
| CH-HuS | Huangshan, Anhui | 30°00′N | 117°59′E | x | x | x | x |  | x | x | x | x | x |  |  |  |
| CH-HZ | Hangzhou, Zhejiang | 30°47′N | 120°03′E |  |  | x | x |  |  |  |  | x | x | x |  |  |
| CH-LiA | Lin'an, Zhejiang | 30°47′N | 120°03′E | x |  |  |  |  | x | x |  |  |  |  |  |  |
| CH-ML | Miluo, Hunan | 28°53′N | 113°12′E | x | x | x | x |  | x | x | x | x |  |  |  |  |
| CH-NJ | Nanjing, Fujian | 24°42′N | 117°3′3E | x | x |  |  |  | x | x | x |  |  |  |  |  |
| CH-WX | Wuxi, Jiangsu | 31°36′N | 120°14′E | x | x | x | x |  | x | x | x | x | x | x |  | x |
| CH-WZ | Wenzhou, Zhejiang | 28°11′N | 120°44′E |  |  | x | x |  |  |  |  | x | x |  |  |  |
| CH-YS | Yangshan, Guangdong | 24°35′N | 112°41′E | x | x | x | x |  | x | x | x | x |  |  |  | x |
| CH-YT | Yingtan, Jiangxi | 28°19′N | 117°03′E | x | x | x | x |  | x | x | x | x | x |  |  | x |
| CH-ZS | Zhangshu, Jiangxi | 28°02′N | 115°25′E | x | x |  | x |  | x | x | x | x |  | x |  |  |
|  |  |  |  |  |  |  |  |  |  |  |  |  |  |  |  |  |

**Table S1 – continued:**

|  |  |  |  |  | | | |  |  | | | | | |  |  |
| --- | --- | --- | --- | --- | --- | --- | --- | --- | --- | --- | --- | --- | --- | --- | --- | --- |
| **ID** | **Location** | **Latitude** | **Longitude** | **Seed collection year** | | | |  |  | **Experiment (seeds)** | | | | |  | **Soil collection** |
|  |  |  |  | **2015** | **2016** | **2017** | **2018** |  | **CG** | **RE** | **SG** | **E** | **C+** | **AC** |  | **E** |
| Introduced populations (in US) | |  |  |  |  |  |  |  |  |  |  |  |  |  |  |  |
| US-AL-1 | Tillman’s Corner, AL | 30°35′N | 88°09′W | x | x | x | x |  | x | x | x | x | x | x |  |  |
| US-FL-4 | Callahan, FL | 30°35'N | 81°47'W | x | x | x | x |  | x | x | x | x | x | x |  |  |
| US-GA-1 | Hutchinson Island, GA | 32°06′N | 81°06′W |  | x | x | x |  |  |  | x | x | x | x |  |  |
| US-GA-2 | Sapelo Island, GA | 31°23′N | 81°15′W | x |  |  | x |  | x | x |  |  | x | x |  |  |
| US-GA-3 | St. Simons Island, GA | 31°08′N | 81°22′W |  | x | x |  |  |  |  | x | x |  |  |  |  |
| US-LA-1 | Lake Charles, LA | 30°14′N | 93°09′W | x | x | x | x |  | x | x | x | x | x | x |  | x |
| US-LA-2 | Lafayette, LA | 30°15′N | 92°01′W |  | x | x | x |  |  |  |  | x |  |  |  |  |
| US-LA-5 | Pumpkin Center, LA | 30°28′N | 90°32′W | x |  | x | x |  | x | x |  | x |  |  |  | x |
| US-LA-6 | Iberville, LA | 30°17'N | 91°05'W |  | x |  |  |  |  |  | x | x |  |  |  |  |
| US-MS-1 | Moss Point, MS | 30°26′N | 88°31′W |  | x | x | x |  |  |  |  | x | x |  |  |  |
| US-SC-1 | Limehouse, SC | 32°09′N | 81°06′W | x | x | x | x |  | x | x | x | x | x | x |  |  |
| US-TX-1 | Houston, TX | 29°47′N | 95°02′W | x | x | x | x |  | x | x | x | x |  |  |  | x |
| US-TX-2 | La Marque, TX | 29°22′N | 95°02′W | x | x | x | x |  | x | x | x | x | x |  |  | x |
| US-TX-3 | Jones Creek, TX | 28°58′N | 95°27′W |  | x | x | x |  | x | x | x | x |  |  |  |  |
| US-TX-4 | Lynchburg, TX | 29°47′N | 95°03′W | x | x | x | x |  | x | x | x | x |  | x |  | x |
| US-TX-5 | Port Arthur, TX | 29°53′N | 94°01′W | x | x | x | x |  | x | x | x | x | x |  |  | x |
|  |  |  |  |  |  |  |  |  |  |  |  |  |  |  |  |  |

**Table S2:**

|  |  | | | |  |  |  |  |  |
| --- | --- | --- | --- | --- | --- | --- | --- | --- | --- |
|  | **AM fungi** | | | |  | **Mass** | | | |
| **factors** | **1st order** | **2nd order** | **3rd order** | **4th order** |  | **1st order** | **2nd order** | **3rd order** | **4th order** |
| TVEH | 3910.2 | 3909.4 | 3899.4 | **3891.1** |  | 753.7 | 754.9 | 753.6 | 755.2 |
| TVE | 3913.2 | 3911.7 | 3906.5 |  |  | 748.1 | 747.1 | 747.4 |  |
| THE | 4034.4 | 4030.1 | 4027.9 |  |  | 1368.5 | 1382.2 | 1381.8 |  |
| VEH | 3913.5 | 3914.7 | 3911.7 |  |  | 953.9 | 960.6 | 961.8 |  |
| TVH | 3913.2 | 3911.7 | 3906.5 |  |  | 1000.0 | 1000.2 | 1003.0 |  |
| TE | 4035.8 | 4033.6 |  |  |  | 1363.2 | 1369.3 |  |  |
| VE | 3915.5 | 3915.4 |  |  |  | 949.0 | 946.7 |  |  |
| EH | 4061.5 | 4061.5 |  |  |  | 1712.6 | 1717.0 |  |  |
| TV | 3932.1 | 3932.6 |  |  |  | 739.4 | **737.4** |  |  |
| TH | 4053.2 | 4052.0 |  |  |  | 1364.3 | 1367.7 |  |  |
| VH | 3932.7 | 3934.2 |  |  |  | 943.1 | 946.0 |  |  |
| T | 4054.8 |  |  |  |  | 1359.0 |  |  |  |
| V | 3934.8 |  |  |  |  | 938.0 |  |  |  |
| E | 4061.1 |  |  |  |  | 1707.6 |  |  |  |
| H | 4091.8 |  |  |  |  | 1778.5 |  |  |  |
|  |  |  |  |  |  |  |  |  |  |

**Table S3:**

|  |  |  |
| --- | --- | --- |
|  | **AM fungi** | |
| **Fixed Effect** | **DF** | **P** |
| Origin | 1,15 | **0.0065** |
| Chemical | 2,362 | **0.0007** |
| Origin × chemical | 2,362 | 0.8829 |
| Concentration (chemical) | 2,362 | 0.2811 |
| Origin × concentration (chemical) | 2,362 | 0.6711 |
|  |  |  |
| **Random effect** |  | **P** |
| Population (origin) |  | 0.1009 |
|  |  |  |

**Table S4:**

|  |  |  |
| --- | --- | --- |
| **Fixed effects** | **DF** | **P** |
| Origin | 1,11 | **0.0028** |
| Charcoal | 1,123 | **<0.0001** |
| Origin 🞨 charcoal | 1,123 | **0.0019** |
|  |  |  |
| **Random effect** |  | **P** |
| Population (origin) |  | 0.0880 |
|  |  |  |

**Table S5:**

|  |  |  |
| --- | --- | --- |
| **Fixed effects** | **DF** | **P** |
| Origin | 1, 20 | **0.0002** |
| Medium | 1, 81 | **0.0008** |
| Origin × medium | 1, 81 | 0.3004 |
| Plant_age (medium) | 2, 266 | **<0.0001** |
| Origin × plant_age (medium) | 2, 266 | 0.7694 |
| Germ_time | 3, 1101 | **<0.0001** |
| Germ_time × medium | 3, 1101 | 0.9237 |
| Germ_time × origin | 3, 1101 | 0.9564 |
| Germ_time × origin × medium | 3, 1101 | 0.9244 |
| Germ_time × plant_age (medium) | 6, 1101 | 0.0087 |
| Germ_time × origin × plant_age (medium) | 6, 1101 | 0.9989 |
|  |  |  |
| **Random effects** |  | **P** |
| Population (origin) |  | **0.0086** |
| Plant_ID (origin × population × medium) |  | **0.0191** |
| Dish_ID (origin × pop × medium × plant_ID × plant_age) |  | **0.0302** |
|  |  |  |

**Table S6:**

|  |  |  |
| --- | --- | --- |
| **Effect** | **DF** | **P-value** |
| Treatment | 7,109 | **<0.0001** |
| Block | 1,109 | **0.0010** |
| Treatment × block | 7,109 | 0.0791 |
| Time | 3,327 | **<0.0001** |
| Time × treatment | 21,327 | 0.9642 |
| Time × block | 3,327 | 0.5528 |
| Time × treatment × block | 21,327 | 0.9999 |
|  |  |  |
